# Development of experimental GBS vaccine for mucosal immunization

**DOI:** 10.1371/journal.pone.0196564

**Published:** 2018-05-04

**Authors:** T. Gupalova, G. Leontieva, T. Kramskaya, K. Grabovskaya, E. Bormotova, D. Korjevski, A. Suvorov

**Affiliations:** 1 Institute of Experimental Medicine, Saint-Petersburg, Russia; 2 Saint Petersburg State University, Saint-Petersburg, Russia; Universita Cattolica del Sacro Cuore, ITALY

## Abstract

*Streptococcus agalactiae*, or group B streptococcus (GBS), is an important pathogen as it is the leading cause of neonatal deaths due to sepsis, meningitis or bacterial pneumonia. Although the development of an effective and safe GBS vaccine is on the agenda of many research labs, there is no GBS vaccine on the market yet. In the present study we attempted to engineer a live vaccine strain based on Bac, a surface protein of GBS, incorporated into a surface fimbrial protein of probiotic Enterococcus. The resulting strain induced specific systemic and local immune responses in mice and provided protection against GBS when administered via the intranasal, oral or intravaginal immunization routes.

## Introduction

*Streptococcus agalactiae* or group B streptococcus (GBS) is the leading cause of death in neonates due to sepsis, meningitis or bacterial pneumonia. Infants are exposed to the bacteria when they pass through the birth canal of the mother, an often asymptomatic carrier of GBS. GBS can also cause miscarriage, intrauterine fetal damage, puerperal sepsis, and other conditions. GBS is increasingly seen as the causative agent of urogenital infections in adults, as well as septic processes in the elderly. Despite the effectiveness of penicillin prophylaxis during the early onset of the infection, antibiotics are useless in preventing the late onset of the disease in neonates. Recently, probiotic treatment of the carriers and infected infants was found to alleviate the disease; however, probiotics on their own rarely ensured complete eradication of the pathogen. This makes GBS vaccine development an effective approach for prophylaxis.

Two different strategies can be used in the development of modern vaccines for the prevention of GBS infection: making polysaccharide conjugate vaccines or making recombinant protein vaccines, which include immunogenic domains of surface bacterial proteins.

A number of multivalent conjugate vaccines based on GBS polysaccharide antigens were constructed, each corresponding to the main capsular serotypes of the bacteria [1]. Recently a trivalent group B streptococcus vaccine was successfully evaluated in a phase 1b/2 trial [2]. However, the experience with pneumococcal polysaccharide vaccines proved that vaccines targeting only a limited number of polysaccharide serotypes leads to rapid shift in the pneumococcal serotype dynamics [3]. This fact reveals a limitation of serotype-specific vaccines and offers insights that may facilitate alternative strategies including usage of vaccines based on immunogenic surface expressed proteins.

Previously it has been shown that GBS surface proteins can also serve as components of a vaccine effective against GBS infection. Preventive vaccination with recombinant proteins corresponding to immunogenic portions of streptococcal surface proteins provided protection of laboratory animals from infections caused by different serotypes of GBS [4–8].

Usually, the effective immunization with protein or polysaccharide vaccines requires two or three subcutaneous or intramuscular injections with an adjuvant. However, this may be associated with serious complications and requires additional organizational efforts and financial resources. These vaccines are based on the appearance of specific circulating IgG at high concentrations, not necessarily at the ports of entry for the infection, which can be an unnecessary burden for the host’s immune system. An alternative to the conventional vaccines is the use of mucosal vaccines which can be as effective as traditional ones. Recently mucosal vaccine based on inactivated GBS was found to be immunogenic and protective [9].

Mucosal vaccines can typically be administered on different mucosal surfaces: orally, intra-vaginally, or by inhalation [10]. The main advantage of live vaccines is that they can be administrated only once and activate all components of the immune system, inducing a balanced immune response at the natural ports of entry for the infection and mimicking the natural infection. Vaccination with live vaccines is often used by health care systems of different countries, but in many cases attenuated viruses or bacteria may return to the virulent form.

This safety issue can be resolved by basing the live vaccines on bacterial probiotic strains. Probiotics are live bacteria that have a generally beneficial effect on the human body (usually, lactic acid bacteria are used as probiotic strains). It was found that some probiotic strains not only have antagonistic activity and the ability to restore the microbiota, but are effective non-specific stimulators for the production of specific antibodies to various infections [11, 12]. Recently, bacterial probiotics have been used as vectors with plasmid constructs of the antigens of pathogenic bacteria [13].

However, the probiotic strains with recombinant plasmids lack stability due to spontaneous plasmid loss. The present approach was based on integration of heterologous DNA into the structure of the chromosomally located surface protein gene of the probiotic without disturbing the open reading frame. For this purpose we used a fragment of the GBS Bac gene encoding for IgA binding region. This protein was previously shown as a potent vaccine antigen [4]. Bac protein expressed in a limited number of GBS serotypes (Ia, Ib, II, IV, IX) has been proven to be associated with the most virulent strains [14].

The aim of the present study was to develop a method of creating a live vaccine based on a probiotic strain, able to induce the appearance of pathogen-specific antibodies due to inclusion of the antigen of the bacterial pathogen in the structure of the pili protein gene. For this purpose Bac protein DNA was integrated into the gene coding for the fimbrial protein D2 of *E*. *faecium* L3.

## Materials and methods

### Ethics statement

All the animal experiments were carried out under the guidelines of the “Rules of Laboratory Practice” of the Ministry of Health of the Russian Federation N° 708. The study was approved by the Local Ethics Committee for Animal Care and Use at the Institute of Experimental Medicine, Saint-Petersburg, Russia. Non-terminal procedures were performed under ether anesthesia. Animals were euthanized by CO2 inhalation, and all efforts were made to minimize suffering of the animals. The health status of the live vaccine challenged mice was monitored and recorded once a day for ten days post last vaccination. No animal showed any signs of illness following vaccine strain infection. No animals died (without euthanasia) as a result of the experimental procedures.

### Bacterial cultures

*E*.*coli* DH5α strain was obtained from the strain collection of the Institute of Experimental Medicine and used as the recipient in transformation experiments. Bacteria were grown in Luria Broth medium at 37°C with constant shaking.

*Streptococcus agalactiae* serotype Ibc (strain H36) was obtained from the collection of the Institute of Experimental Medicine, St. Petersburg. Bacteria were cultured in THB medium (Todd-Hewitt broth) (HiMedia, India) for 24 hours at 37°C under aerobic conditions, washed three times with PBS by centrifugation at 3500 rpm for 20 minutes, and concentrated as needed. The resulting suspension was used for infection.

A culture of the original *E*. *faecium* L3 strain or genetically modified *E*. *faecium* L3-Bac+ was grown in sterile THB (c 0.5% yeast extract) and incubated at 37°C for 24 hours. Bacteria were washed three times by centrifugation at 3500 rpm for 20 minutes. The bacterial sediment was suspended in PBS to the desired concentration. The resulting suspension was taken for vaccination of mice.

### Method of bacterial quantification

10 μl of 10-fold sample dilutions are dropped on the surface of 5% blood agar medium and are incubated for 24 hours at 37°C. Bacterial development is controlled in each drop. The isolated bacteria are counted and the number of colonies is used as a measure of the number of CFU (colony forming units) in that known dilution. By extrapolation, this number is used to calculate the number of CFU per milliliter of original sample. The number of CFUs per ml of sample = the number of colonies X 100 X the dilution factor of the drop counted.

### Making a chimeric protein of the *E*. *faecium* L3 d2 gene and the fragment of the *bac* gene

To use the probiotic strain *Enterococcus faecium* L3 chromosome as a template in a polymerase chain reaction (PCR), the chromosomal DNA was isolated. A P6 plasmid DNA was used as a template in order to amplify a portion of the *bac* gene [15].

DNA fragments corresponding to the fragments of D2 gene from *E*. *faecium* L3, encoding pili protein and the fragment of the *bac* gene were amplified by PCR with Tag polymerase (Ampli Tag, Perkin-Elmer, Cetus, USA) using a thermocycler (BIO-RAD, USA). The oligonucleotide primers used for the reaction are listed in Table 1. As a result of three separate reactions with the primers A1-B1 and C1- D1 for enterococcal DNA, and the primers E1 and F1 for *bac* gene, three fragments of DNA were obtained. The PCR program included denaturation at 94°C—30 sec, primer annealing– 55°C—1 min, and synthesis—72°C—1 min. This cycle was repeated 30 times, after which the mixture was incubated at 72°C for 10 minutes. The PCR products were separated on a 1% agarose gel in a horizontal electrophoresis. Amplified DNA segments were isolated from the agarose QIAquick Gel Extraction Kit (Qiagen, USA).

**Table 1:** Oligonucleotide primers.

Primers	Direction	Nucleotide sequence from 5' to 3'
A1	forward	GGGGTACCCCCGATGAGAGCAGCTGGTATTG
B1	reverse	CAGAATCATTTGTTTCATCAAACAATGCGCCATCATAGTTT
C1	forward	TGGAGCAGGTTGAGAAGGAAGGTTCTGCGCGAGTGATAGAT
D1	reverse	CAACAAGCTTCAAAGCATCGTTGG
E1	forward	TTGATGAAACAAATGATTCTGATG
F1	reverse	TTCCTTCTCAACCTGCTCCA
D2	reverse	CAACAGGATCCAAAGCATCGTTGG
B2	forward	TGAGTGAACCACAGCCAGAA
B3	forward	TCAGCAACGTGTGTCTTGGT
B4	reverse	CGAACCTTTACTTCGGCATC
B5	reverse	GTGATTCCCTTTGCTCTGC

The bold sections in the nucleotide sequences indicate the sites of restriction endonucleases used to create the design.

### Preparation of a fusion gene ent-bac and cloning

The synthesis of a hybrid DNA fragment was performed with primers A1 and D1 PCR using the program described above, wherein the synthesis time at 72°C was increased to 2 minutes. DNA isolation and analysis of the size of the resulting DNA fragment’s amplified portion was performed as described above. Cloning of the amplified DNA fragment was performed using the set of plasmids pJET1.2 Clone JET ™ PCR Cloning Kit. The ligation mixture was used to transform *E*.*coli* DH5α in a heterologous system. The medium for selection of transformants contained 100 ug / ml ampicillin. Hybrid (*ent-bac*) DNA was subcloned into a suicidal plasmid pT7ermB with the gene of resistance to erythromycin. For this purpose, an amplification employing the primers A1 and D2 was carried out. The PCR product and the plasmid were digested with pT7ERMB enzymes *Bam*HI and *Kpn*I. The hydrolysis products were separated by electrophoresis in 1% agarose gel and purified from the agarose using the set QIAquick Gel Extraction Kit (Qiagen, USA), then ligated and transformed into the *E*.*coli* DH5α heterologous system. The LB medium for selection of *E*.*coli* transformants contained 500 mcg/ ml of erythromycin. In order to check the construct, plasmid DNA *pent-bac* was used as a template in a PCR with primers B3 and B4 (Table 1). The amplification product was purified from agarose gel and sequenced.

### Analysis of the nucleotide and amino acid sequences

The nucleotide sequence of the DNA was determined by the Research and Production Company Syntol. The amino acid sequence was determined on the basis of the nucleotide sequence using the computer program ExPASy translate tool [16].

### Electroporation of enterococci

In the first step of the transformation process *Enterococcus faecium* L3 culture was cultivated in 3 ml of THB and grown overnight at 37°C; then, 1 ml of the culture was resuspended in 50 ml of THB broth and grown to an optical density 0.3 at 650 nm. After that, the culture was placed in ice and then washed three times in 20 ml of 10% glycerol at 4°C. The resulting bacterial pellet was suspended in 0.5 ml of sterile glycerol solution and transferred into Eppendorf tubes, 50 μl in each tube. After the DNA (300 ng) was added, the enterococci were electroporated in a cuvette with a 1 mm electrode spacing at 2100 V. The pulse duration was 4.5 milliseconds. After the current was discharged, 1 ml of THB was added to the cuvette, incubated for 1 hour and plated on selective media containing 10 μg / ml of erythromycin. Transformants were expected to appear in 24 hours.

### Confocal microscopy

A DNA-binding fluorescent dye, SYTOX Green (green) from the set SelectFX (Invitrogen, USA), was used for the coloring of *E*. *faecium* L3.

Immunohistochemical detection of protein Bac in Enterococcal clones was carried out with human serum IgA (Sigma) conjugated with horseradish peroxidase (HRP). *E*.*faecium* L3-Bac+ was incubated with IgA-HRP conjugate for 30 minutes at 25°C. In order to carry out further fluorescence imaging in a confocal laser microscope, a peroxidase was detected by reaction with goat anti-HRP IgG, conjugated with the fluorochrome Cy3 (red) (Jackson ImmunoResearch, USA). The obtained slides were analyzed by confocal argon laser microscope LSM 710 (Zeiss, Germany) (488 nm) or solid state (561 nm) lasers.

### Detection of enterococcal clones expressing Bac

Enterococcal clones were spotted in doubles on the LB agar with 500 mcg/ml of erythromycin. Colonies from one of the plates were transferred to nitrocellulose membrane and lysed in solution containing 0.2 N NaOH, 0.1% SDS and 0.5% β- mercaptoeathanol.

The membrane was washed in PBS and incubated first for one hour at room temperature in a blocking solution (2 parts of 3% milk and one part of PBS), and then for one hour at room temperature in a blocking solution with peroxidase-labeled IgA. The membrane was washed in a blocking solution and then in PBS. Peroxidase activity was determined (TMB, Sigma, DNA). Bac+ clones, which bind IgA, were also tested by PCR with the primers to *bac* gene.

### Immunization of mice with live probiotic vaccine

The 8- to-10-week-old female, outbred mice were provided by the laboratory breeding nursery of the Russian Academy of Sciences (Rappolovo, Leningrad Region). Mice were housed in groups of twenty in 400х250х200 mm cages (Plastpolymer, Russia), maintained under standard conditions and given ten days to acclimate to the housing facility. All animals were housed in a special pathogen-free facility, fed autoclaved food and water ad libitum. At the start of the experiments animals weighed (mean±SD) 20,0±2,0 grams. Immunogenic properties of the *E*.*faecium* L3-Bac+ vaccine strain were tested in three modes of administration. Mice were distributed in groups in a random way. In the case of intravaginal immunization (n = 60), *E*. *faecium* L3-Bac+ was administered on days 1, 2, 3, 4, and 5 in a volume of 20 μl PBS at a dose of 2 × 10^7^ CFU / mouse; intranasal immunization (n = 160) *E*. *faecium* L3-Bac + was administered on days 1, 2, 21, 22, 42 and 43 from the start of the experiment in a volume of 50 μl of PBS at a dose of 1.5 x 10^8^ CFU / mouse. For the oral immunization (n = 40) *E*. *faecium* L3-Bac+ was administered in drinking water on days 1, 2, 3, 6, 7, 22, 23 and 24 from the start of the experiment at a dose of 2 × 10^8^ CFU/mouse. Control groups were vaccinated with the E. faecium L3-Bac(-) negative strain in the same way. The number of mice in control and experimental groups was equal.

Blood samples were taken from the submaxillary vein, and vaginal lavages were obtained by washing the vaginal cavity with 50 μl PBS. After sampling PMSF was added to the lavage to a final concentration of 1 mM. To determine specific nasal secretory IgA, mice were injected intraperitoneally with 0.1 ml of a 0.5% pilocarpine solution, and after 1 to 2 minutes, immediately after the onset of increased salivation, 50 μl of secretions were collected. The PMSF protease inhibitor was added to the samples for a final concentration of 1 mM.

There were no less then three days between blood, nasal or vaginal sampling and infection.

### Specific immunoglobulins detection

Specific IgG and IgA levels were determined by ELISA in 96-well ELISA plates (Nunc) coated with the protein P6 (2μg/ml) overnight at 4°C. A series of twofold dilutions of the sample(100 μl) was added to duplicate wells and incubated for 1 h at 37°C. HRP-labeled goat anti-mouse IgA and IgG antibodies (Sigma) diluted in a blocking buffer according to the manufacturer’s instructions were added (100 μl/well). After incubation at 37°C for 1 h, the plates were developed with TMB substrate (BD Bioscience) according to the manufacturer’s instructions. The Ig concentration was measured with reference to standard curves (S1 Protocol) using the known amount of IgG or IgA (Sigma).

### Study of the protective efficacy of vaccination

In order to investigate the specific protective effectiveness of the immune response, mice were infected with GBS (H36 Iac) intravaginally at a dose of 10^9^ CFU/mouse, intraperitoneally at a dose of 0.5 x 10^7^ CFU/ mouse, or intranasally at a dose of 1.5 x 10^8^ CFU/ mouse. Vaginal lavages and spleen or lung tissues were collected after 2, 5 and 24 hours.

Lung and spleen tissues were harvested and homogenized in PBS using a Retsch MM-400 ball vibratory mill.Vaginal lavages were examined directly without any processing. Serial 10-fold dilutions of homogenates were made in PBS and aliquots of the dilutions were plated on dense nutrient medium (Columbia agar with 5% human erythrocytes). Plates were incubated at 37°C for 14–16 hours before the colonies were counted under a microscope. The bacterial burden in CFU per organ was calculated and expressed as log10.

### Statistical analysis

Data was processed using Statistica software, version 8.0. (StatSoft, USA). Means and standard errors of the means were calculated to represent IgA and IgG concentration and bacterial number. ANOVA test was used to compare two independent groups. The p-value(s)<0.05 were considered to be statistically significant.

## Results

**Fig 1:**
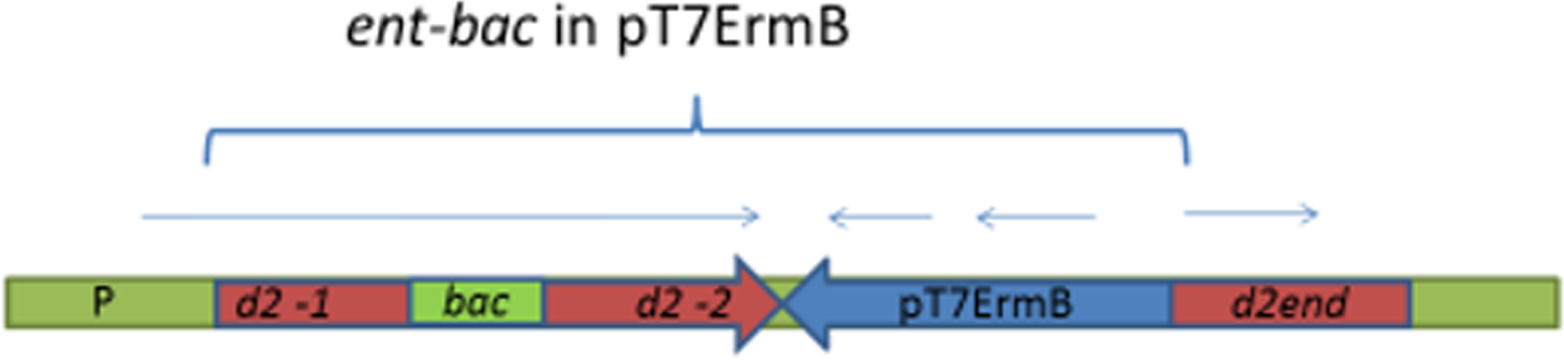
Integration of the plasmid pT7ermB with the *ent-bac* into the chromosome of the strain *E*. *faecium* L3. P-promoter of the gene *d2*; *d2*-1-a region of the *d2* gene encoding for N- terminal part of D2 protein; *bac*- a fragment of the *bac* gene, encoding for IgA binding; D2-end–end of the *d2* gene encoding for the C terminus of D2 protein; pT7 ErmB—integrative plasmid. Arrows correspond to the open reading frames in the integrated element. The entire integrated element *ent-bac* with plasmid pT7ErmB is shown in brackets.

The fusion gene *ent*-*bac* in the E.coli plasmid pJET1.2 was obtained using PCR with the DNA primers listed above. The primers were designed so as to allow the resultant fragment to be cloned with restriction endonucleases and to retain the open reading frame with no stop codons. As a result of two consecutive steps of PCR, separate parts of the chimeric DNA structure were obtained, and then a merged DNA fragment with a *bac*–gene fragment in the middle of enterococcal gene encoding for pili was obtained (S1 and S2 Figs). The insert size was 1,8 kb. Following the cloning, several transformants with the proper insert were selected. One *E*.*coli* clone, which was able to bind IgA, was selected for further studies. Hybrid DNA (*ent-bac*) from this clone was re-cloned in suicidal plasmid pT7ermB, which is unable to replicate in gram positive bacteria. The resultant *E*.*coli* clones were selected and checked for IgA-binding activity. The nucleotide sequence of the insert corresponded to two fragments of the D2 gene from Enterococcus and a fragment of *bac* gene (*ent-bac*) in the middle (Fig 1).

An integrative plasmid with *pent-bac* DNA was used for the electroporation of the enterococcal strain *E*. *faecium* L3 as described in materials and methods. Erythromycin resistant enterococcal transformants were taken the next day to be tested for IgA binding properties provided by the fragment of the protein Bac. Bac positive clones were selected and tested in a PCR reaction, with primers corresponding to the *bac* gene sequence (primer B5) and chromosomal DNA sequence of enterococci (primer B2) to confirm chromosomal integration plasmid DNA p*ent-bac*. The amplification product was purified from agarose gel (S3 Fig) and sequenced (S4 Fig).

This clone of enterococci with *bac* gene was designated *as E*. *faecium L3* Bac + and selected as a vaccine preparation for further study.

**Fig 2:**
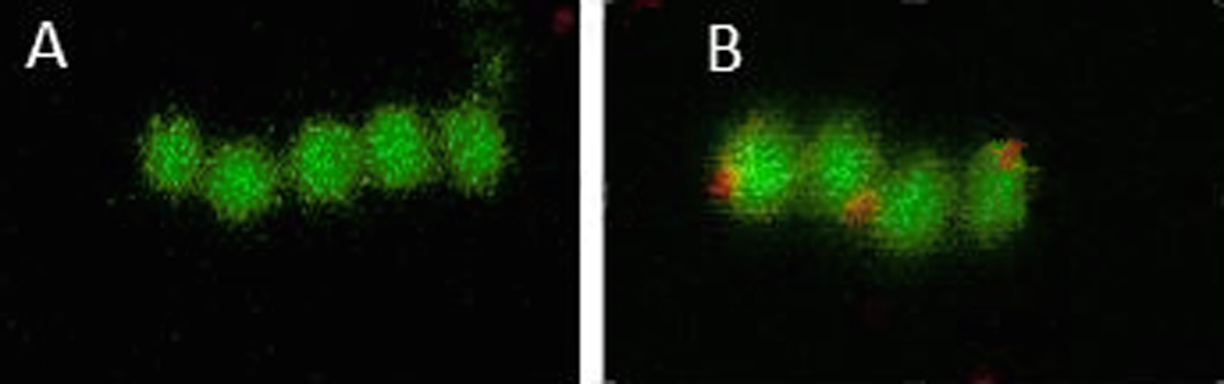
Confocal microscopy of the original enterococcal strain *E*. *faecium* L3 and its derivative with the fragment of streptococcal *bac* gene using an HRP labeled IgA. A- *E*. *faecium L3*, *B- E*. *faecium L3* Bac +.

To verify the expression of the chimeric gene in *E*. *faecium L3* Bac +, bacteria were analyzed by confocal microscopy using an HRP labeled IgA (red). The results provided evidence of specific interaction of the bacteria *E*. *faecium L3* Bac + with IgA which was manifested by the appearance of additional red fluorescence on the surface of bacteria (Fig 2).

The fact that the human serum IgA binds to the surface of the genetically modified enterococcus demonstrated the emergence of IgA-binding protein Bac on the cell surface (Fig 2B). The original *E*.*faecium* L3, used as the control, did not demonstrate any significant fluorescence (Fig 2A).

**Fig 3:**
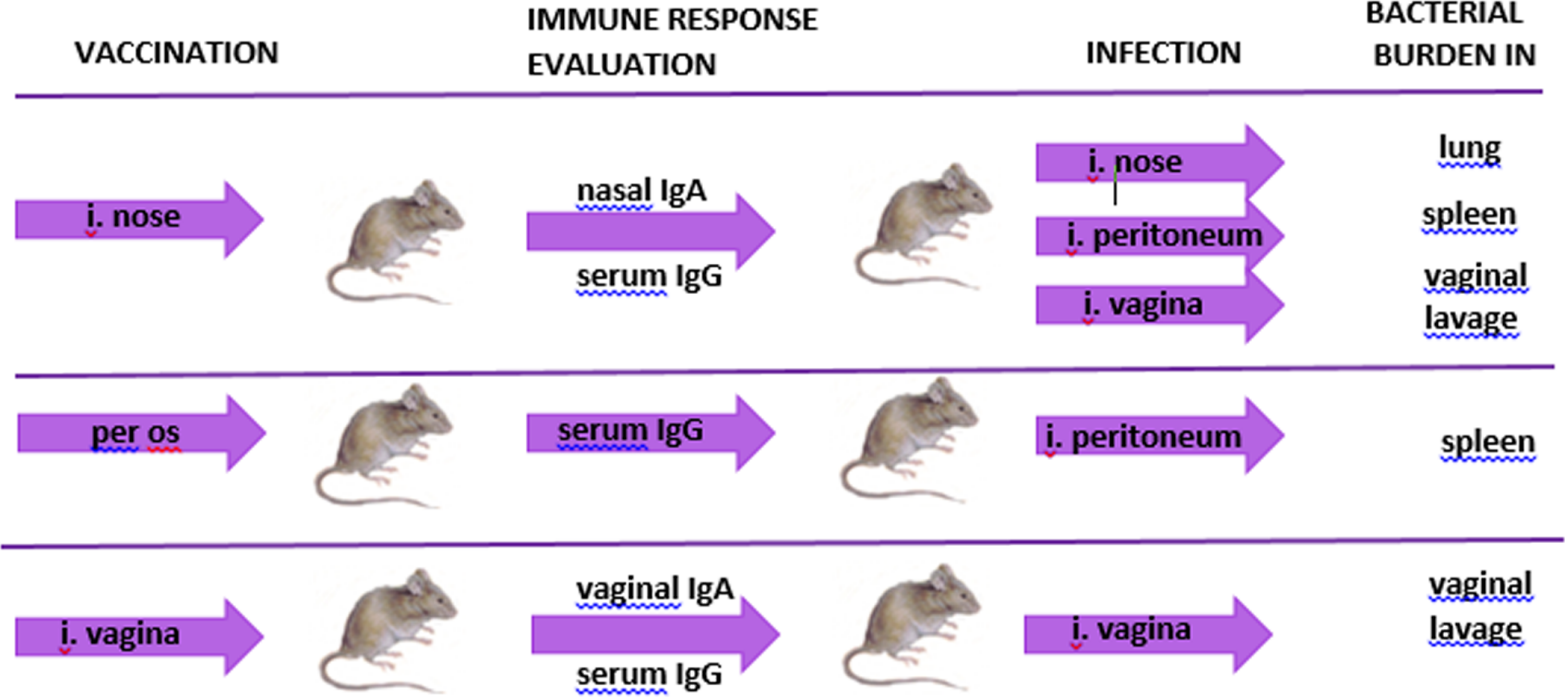
Immunological study and analysis of *E*. *faecium* L3 Bac +protective properties. General setup.

It was necessary to find out whether *E*. *faecium L3* Bac + stimulated a Bac-specific immune response after application through mucosa, and whether the immune response was sufficient to protect mice against GBS infection. The general experimental setup is presented in Fig 3.

The experimental groups of mice were vaccinated through different mucosal surfaces, with the vaccine applied through the nose, mouth or vagina. Secretory and systemic immune responses were measured over the course of vaccination. Immune mice were infected with the GBS strain H36 (Iac) by different routes for each method of vaccination. The microbe burden was examined in lung, spleen or vaginal lavages according to the type of infection present: nasal, peritoneal or vaginal.

**Fig 4:**
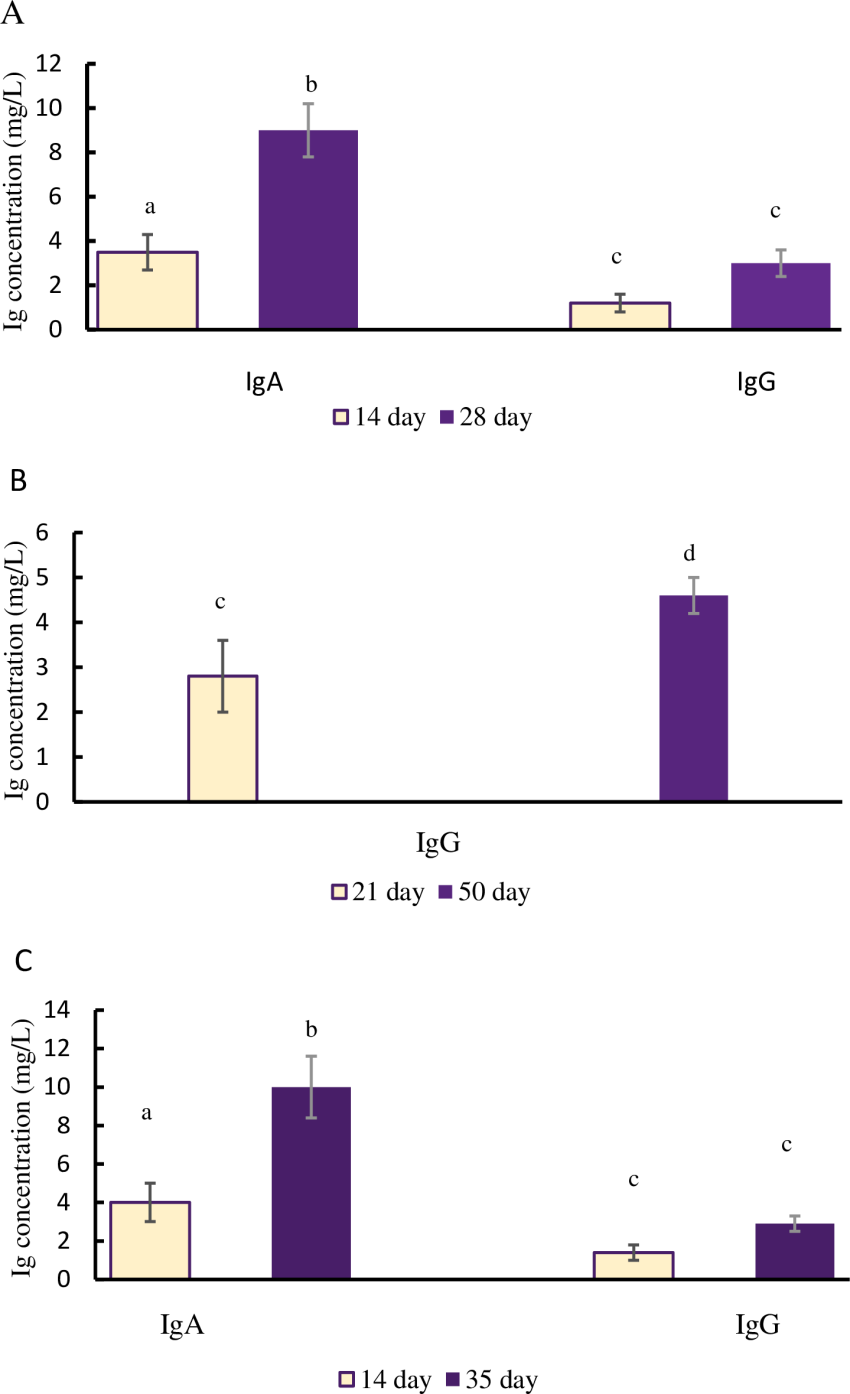
The immune response in mice after vaccination with live vaccine. Mice were vaccinated vaginally (A), per os (B) nasally, (C) with *E*. *faecium* L3Bac +. The Bac-specific secretory IgA and/or serum IgG levels were measured after vaccination. Each point on the chart represents the average value from 10 measurements. In controls, the concentration of specific mucosal IgA and serum IgG did not exceed 3 mg/L. Means with different letter differ significantly (p<0,05). Symbols a-b are valid for IgA, c-d for IgG.

The immunogenic properties of the vaccine strain of *E*. *faecium*L3 Bac+ were studied after intravaginal, oral, and intranasal immunization of the mice. Over the course of the immune response expression, the content of Bac-specific antibodies was measured in serum as well as nasal and vaginal lavages (Fig 4).

All methods used for the mucosal immunization of mice with the modified probiotics were able to stimulate a specific immune response. Mice treated with *E*. *faecium* L3 Bac+ after intravaginal or intranasal immunization were demonstrated active growth of Bac-specific secretory IgA (p<0,05) in the vaginal and nasal lavages. A gradual accumulation of serum IgG antibodies (Fig 4A and 4B) was registered. After oral immunization, a significant increase of specific IgG levels (p<0,05) in serum was recorded on day 50 (Fig 4C).

**Fig 5:**
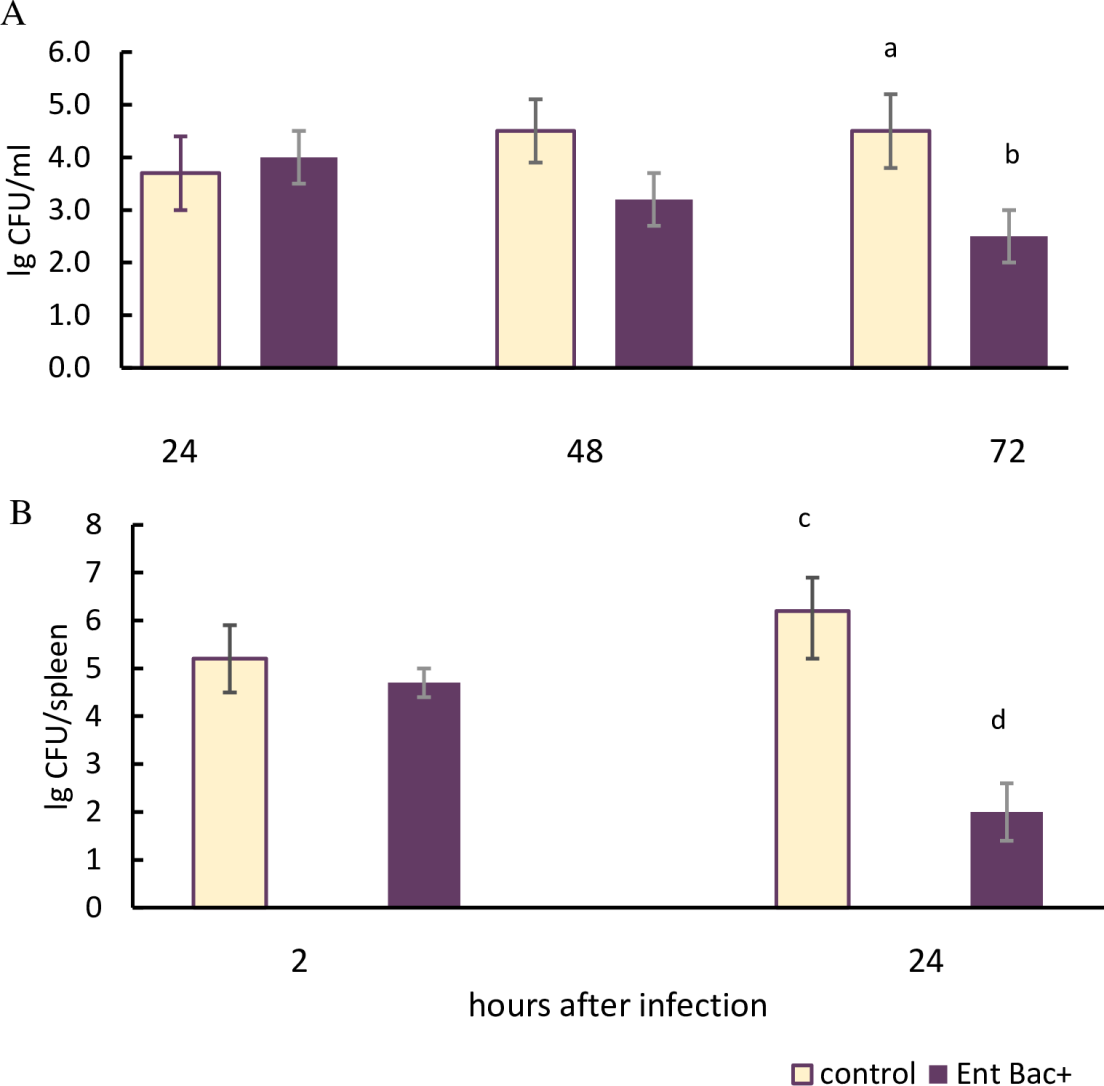
The bacterial count at different stages of GBS infection in mice after vaginal and oral vaccination. Vaginally vaccinated immune mice (n = 60, 10 mice per each point) were infected intravaginally with GBS (H36 Iac) (A). At the same time, orally vaccinated immune mice (n = 40, 10 mice per each point) were infected intraperitoneally with the same strain (B). After infection, the bacterial burden in CFU within the vaginal cavity (A) and the spleen (B) was calculated and expressed as log10. (A) Means with letter a and b differ significantly (p<0,05).(B) Means with letter c and d differ significantly (p<0,05).

**Fig 6:**
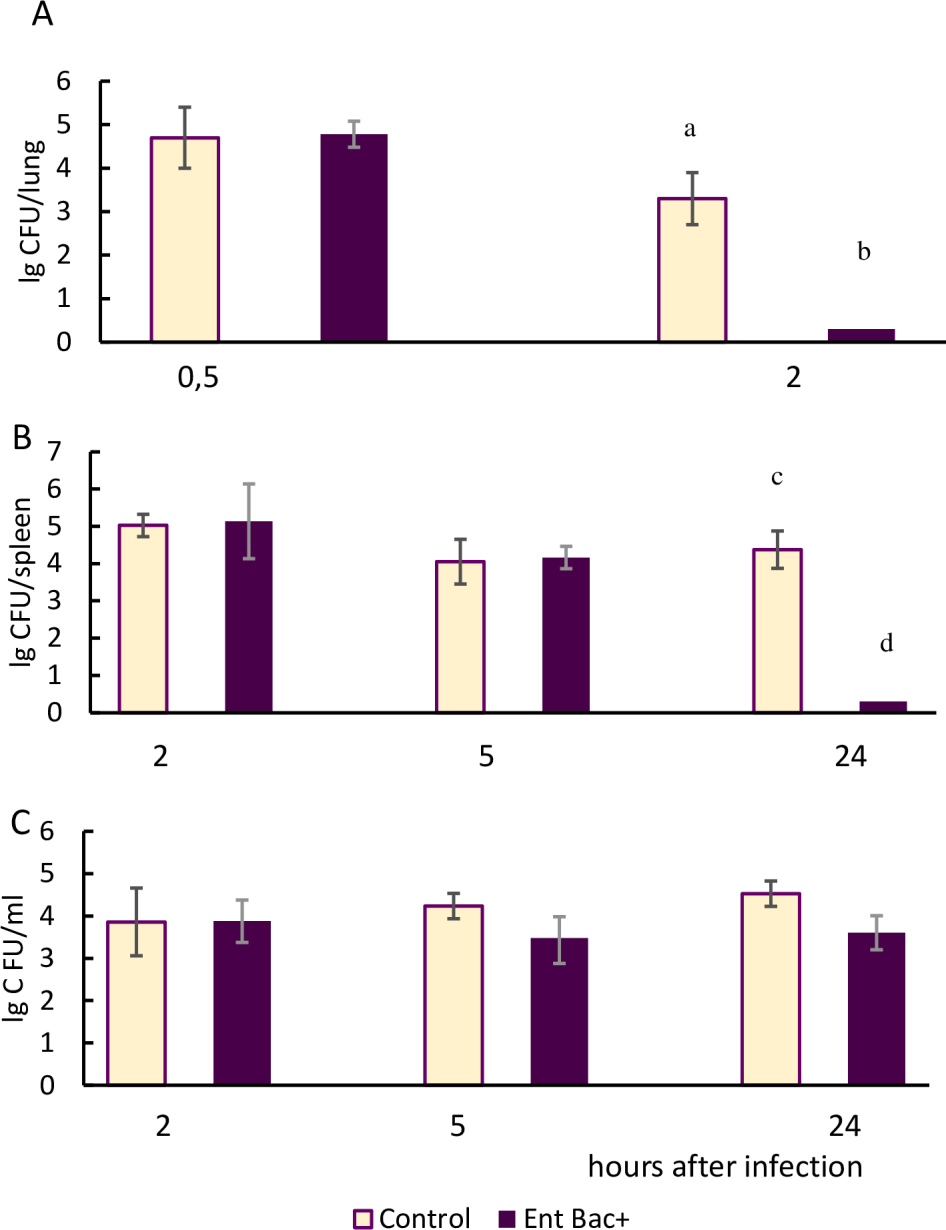
The bacterial count at different stages of GBS infection in mice after nasal vaccination. Nasally vaccinated immune mice (n = 160, 10 mice per each point) were infected with GBS (H36 Iac) by intranasal (A), intraperitoneal (B) and vaginal (C) routes. After infection, the bacterial burden in CFU within lungs (A), spleens (B), and vaginal lavages (C) was calculated and expressed as log10. (A) Means with letter a and b differ significantly (p<0,05) (B) Means with letter c and d differ significantly (p<0,05).

To evaluate the protective efficacy of a specific immune response, vaccinated mice were infected by group B Streptococcus strain H36 (Iac) containing the protein Bac. The infection of immune animals was carried out after the end of the vaccination courses using a variety of bacterial application routes. In order to assess the rate of protection, we checked the level of bacterial burden in CFU per organ at the different stages of bacterial infection. Mice administered with original strain of *E*. *faecium*L3 (Bac-) in PBS by an equivalent route served as the control (Figs 5 and 6).

In the group of mice vaccinated intravaginally, vaginal GBS infection was produced (Fig 5A).

In 48 hours after infection, a reduction of the bacterial count in the vaginal lavages of the immune mice compared to the control (p = 0,12) was registered, and 72 hours after infection, the differences became significant (p<0,05). In the control group of animals the bacterial count in the lavages did not change after 72 hours.

Mice vaccinated orally via the mucosa of the digestive tract were infected intraperitoneally with group B streptococcus (Fig 5B). 24 hours after infection a significant reduction in the GBS burden within spleens was registered in immune mice. Within the spleens of control mice the bacterial count remained at a constantly high level.

In order to test the probiotic vaccine introduced intranasally, three different variations of GBS infection (intranasal, intraperitoneal and vaginal) were used (Fig 6). Taking into account the relatively low virulence of GBS to the mice after nasal infection, we monitored the bacterial burden of the Bac + GBS strain during the first 2 hours of GBS application.

Two hours after intranasal (Fig 6A) and 24 hours after intraperitoneal (Fig 6B) infection, streptococcus were absent in the lungs and spleens of immune mice, whereas the control animal tissues were contaminated by GBS in high amounts. 24 hours after the vaginal GBS challenge, the vaginal lavages of the immune mice contained significantly fewer bacteria than the control (Fig 6C).

The data received indicated that the intranasal route of vaccination with probiotic live vaccine provided protection against GBS infection not only in the respiratory tract area, but also on the mucosal surfaces of the vagina and in the peritoneal cavity. Thus, the vaccination of mice with the live probiotic vaccine *E*. *faecium* L3-Bac*+* through the mucosal surfaces of the vagina, the respiratory tract, or the digestive tract resulted in the accelerated elimination of the infectious agent from different body sites (compared to the control).

## Discussion

At present, when widely spread resistance of bacterial pathogens to antibiotics significantly jeopardizes the ability of clinicians to treat infections, vaccine prophylaxis of bacterial infections has become essential. However, there are several factors which are impeding the development of bacterial vaccines. First, though using attenuated live bacterial pathogens is possible, it is never 100% safe. Another concern is the immunopathological reactions which might be induced by bacterial surface structures, making the cross-reactive antibodies active against human tissues [17]. Important bacterial pathogens such as *S*. *agalactiae*, *S*. *pneumonia*, *Staphylococcal aureus*, and *Enterococcus faecalis* are a part of the natural microbial communities of many healthy individuals. Therefore, the advantages of the complete eradication of these bacteria (on the species level) from the different body sites are debatable. That is why it is preferable to address the specific immune response to non-pathogenic microbes expressing certain virulence factors. At the same time, intramuscular or subcutaneous vaccination, especially in the case when several injections are needed, significantly decreases the compliance of the population to properly performed vaccination. Using probiotics as antibacterial agents is an old but well-proven strategy. Nevertheless, in most cases, probiotics are less specific and can positively influence systemic immunity. In this respect the use of a probiotic strain which, retains both all features beneficial to health and the ability to specifically induce an immune response against certain bacterial virulence factors, appears to be a reasonable prophylactic approach. Many probiotic-based strategies of delivering the vaccine antigen onto bacterial surfaces have been developed so far [18].

However, in most studies genes, encoding for the vaccine antigen, were introduced into plasmids, usually lacking stability [19].

Most common probiotic strains used as mucosal vaccine carriers belong to the lactobacillus or lactococcus species, many of which grow poorly in the oxygen-rich environment. In this study we used the enterococcal probiotic strain as the vaccine antigen vector due to its ability to proliferate in a broad range of physiological conditions. In order to create a stable live vaccine against GBS, an approach based on the introduction of the GBS gene into the chromosomal DNA of the probiotic *Enterococcus faecium* L3 was utilized [20].

The probiotic strain of *E*. *faecium* L3 shows pronounced antagonistic activity against a variety of pathogenic Gram-positive and Gram-negative bacteria. It can restore bowel microbiocenosis in dysbiotic conditions after antibiotic treatment, and can have an immunomodulatory effect on the host organism, promoting the expression of IL-10 [21]. The genome of *E*. *faecium* L3 was sequenced completely and proved to be free from virulence genes or actual antibiotic resistance genes typical for clinical strains of enterococci [22]. The strain *E*. *faecium*L3, as well as many other Gram-positive bacteria, possesses pili or fimbriae–surface structures which are protein cylinders 1–1.5 mm in length and with a diameter of 7–10 nm [23]. These protein polymers on the surface of bacteria are the strands of subunits, which are made up of chains of one or several pilin proteins. Pili are highly immunogenic structures which are under strong selective pressure from host innate immunity [24]. Pili of enterococci were considered to be good candidates for the insertion of the fragments in the development of the vaccines as they are exposed on the cell surface (not shielded by a capsule).

In the present work we tested the possibility of modifying a pilis of a probiotic enterococcal strain by introducing an antigenic protein fragment from another bacteria–*S*.*agalactiae****–***into the middle of the pilus protein D2.

For this purpose, we used mice, which are a widely used experimental model in the study of GBS infection [25, 26]. In present study as well as in our early tests of the protective efficacy of recombinant GBS proteins the experiments were carried out on the model of outbred mice [27–29].

The recombinant probiotic strain was able to express the chimeric protein on the surface and produce Bac-specific antibodies in the blood and on the mucosal surfaces. The pathogen was eliminated from the different body sites of mice immunized with a live probiotic vaccine through different mucosal surfaces such as the vagina, the respiratory tract, or the digestive tract significantly faster than from the controls—an effect which might be influenced by the GBS antigen Bac.

*Bac* or *beta antigen C* gene, which was selected for incorporation into the enterococcal genome, encodes a surface protein, which is expressed in several GBS strains of serotypes Ia, Ib, II, V and IX. A distinctive feature of the protein Bac is its ability to bind the Fc-part of human immunoglobulin A (IgA)–the main type of immunoglobulin providing protection against the penetration of microorganisms through the mucosal surfaces. [30]. Also, Bac protein interacts with factor H—blood plasma protein, which when bound to the GBS cell leads to the inactivation of the alternative complement pathway. The ability of the Bac protein to interact with these two components of the human immune system allows GBS to evade the immune response [31]. Bac was selected as a potential component of vaccines due to its prevalence among the virulent GBS strains and its conservative protein structure. Furthermore, it was previously shown that a recombinant derivative of Bac–protein P6 –possesses good immunogenic properties, and its introduction into laboratory animals stimulates the immunity from lethal GBS infection, therefore making it a good vaccine candidate [4].

The restriction sites incorporated into the artificial genetic construct generated in the present study allow the insertion of almost any bacterial gene or genes instead of the *bac* gene. A significant advantage of the present approach is based on the fact that a safe probiotic strain expressing the selected antigen or antigens on the surface can multiply in the mucosa of the vaccinated organism, which dramatically increases the dose of the antigen introduced through the natural ports of entry for the infection. This selective approach will supposedly help to eradicate not a species of the pathogen, but only the strains expressing a certain virulence factor as a target for specific IgG.

## Conclusions

In summary, we have created a probiotic strain which expresses the GBS antigen on the cell surface. Analysis of the blood, serum, and lavages of mice immunized with *Enterococcus faecium* L3- Bac + intravaginally, intranasally, and orally showed that vaccination stimulated the synthesis of Bac-specific IgA and IgG, indicating the development of a local and systemic immune response. In comparison to the control, immunized mice were more resistant to intravaginal, intraperitoneal and intranasal infection with GBS strain H36 carrying protein Bac on the surface. These results allows us to conclude that the genetic modification of the *Enterococcus faecium* L3 probiotic strain provided for the expression of the GBS protein on the surface of enterococcus. This work serves as a successful example of creating a live vaccine for the prevention of GBS infections. The developed process allows for the inclusion of the antigens of any clinically relevant pathogen in the structure of *Enterococcus faecium* L3 pili. The proposed approach opens up the possibility of creating a wide range of live vaccines of varied specificity on the basis of probiotic strains.

## Supporting information

S1 Checklist Development of experimental GBS vaccine for mucosal immunization. (DOCX)

S1 Fig 1% agarose gel electrophoresis pattern of the amplified DNA fragments used for making a chimeric construct. 1–100 bp Ladder DNA marker (100–3000 bp); 2 –the PCR product with the primers A1 and B1; 3 –the PCR product with the primers C1 and D1; 4 –the PCR product with the primers E1 and F1.
(PDF)

S2 Fig 1% agarose gel electrophoresis of the amplified fused DNA fragment. 1—PCR Product («fused» gene) with the primers A1 and D2; 2–100 bp Ladder DNA marker (100–3000 bp).
(PDF)

S3 Fig Amplified DNA fragment with primers B2 and B5. 1–100 bp Ladder DNA marker (100–3000 bp); 2—PCR product with primers B2 and B5
(PDF)

S4 Fig Nucleotide sequence of PCR product obtained after PCR with primers B2 and B5. Primer B2 corresponds to the *E*.*faecium* chromosomal DNA outside the integrative plasmid. Primer B5 corresponds to the streptococcal Bac protein gene.
(PDF)

S1 Protocol Conversion of antibody dilutions and OD values (based on ELISA readings) to antibody concentrations. (PDF)
