# Correction: Development of experimental GBS vaccine for mucosal immunization

**DOI:** 10.1371/journal.pone.0198577

**Published:** 2018-06-01

**Authors:** The PLOS ONE Staff

There is an error in Table 1. Sections within nucleotide sequences from primers A1, D1, and D2 should be bolded. The publisher apologizes for the error. Please see the corrected Table 1 here.

**Table 1:** Oligonucleotide primers.

Primers	Direction	Nucleotide sequence from 5' to 3'
A1	forward	GG**GGTACC**CCCGATGAGAGCAGCTGGTATTG
B1	reverse	CAGAATCATTTGTTTCATCAAACAATGCGCCATCATAGTTT
C1	forward	TGGAGCAGGTTGAGAAGGAAGGTTCTGCGCGAGTGATAGAT
D1	reverse	CAAC**AAGCTT**CAAAGCATCGTTGG
E1	forward	TTGATGAAACAAATGATTCTGATG
F1	reverse	TTCCTTCTCAACCTGCTCCA
D2	reverse	CAACA**GGATCC**AAAGCATCGTTGG
B2	forward	TGAGTGAACCACAGCCAGAA
B3	forward	TCAGCAACGTGTGTCTTGGT
B4	reverse	CGAACCTTTACTTCGGCATC
B5	reverse	GTGATTCCCTTTGCTCTGC

The bold sections in the nucleotide sequences indicate the sites of restriction endonucleases used to create the design.
