# Supplementary material for: Development of experimental GBS vaccine for mucosal immunization
Source: PLoS One. 2018 May 4;13(5):e0196564. doi: 10.1371/journal.pone.0196564 (PMC5935385; doi:10.1371/journal.pone.0196564)

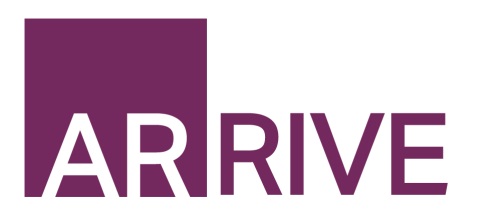


The ARRIVE Guidelines Checklist

Development of experimental GBS vaccine for mucosal immunization

T. Gupalova 1¶, G. Leontieva 1¶ *, T. Kramskaya 1¶, K. Grabovskaya 1&, E. Bormotova 1&, D. Korjevski 1&, A. Suvorov ¶1,2*^1^*

*1. Institute of Experimental Medicine, Saint-Petersburg, Russia*

*2. Saint Petersburg State University, Saint-Petersburg, Russia*

|  | | ITEM | RECOMMENDATION | Section/ Paragraph |
| --- | --- | --- | --- | --- |
| 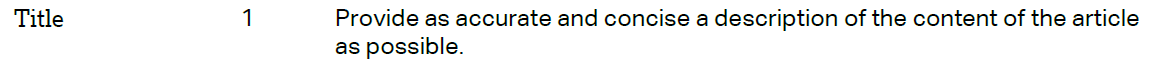 | | | Title |  |
| 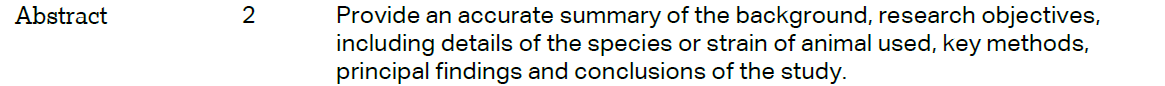 | | | Abstract |  |
| INTRODUCTION | | |  |  |
| 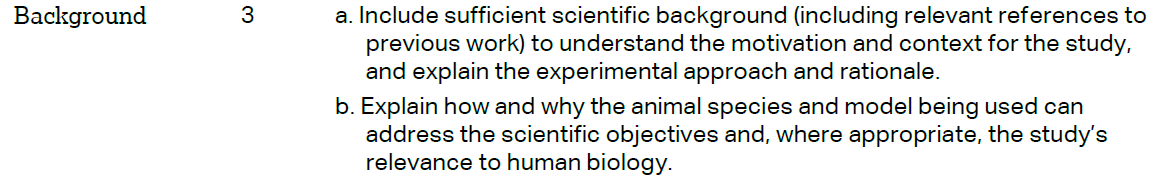 | | | Paragraphs 1-6  Paragraphs 7-8 |  |
| 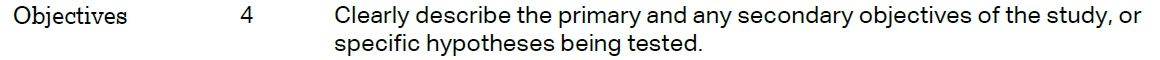 | | | Paragraph 9 |  |
| METHODS | | |  |  |
| 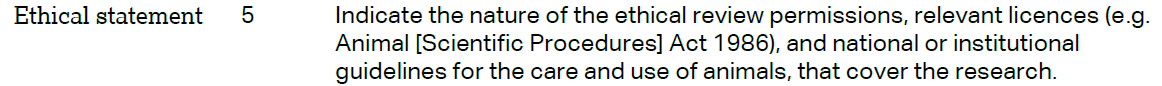 | | | Paragraph 1 |  |
| 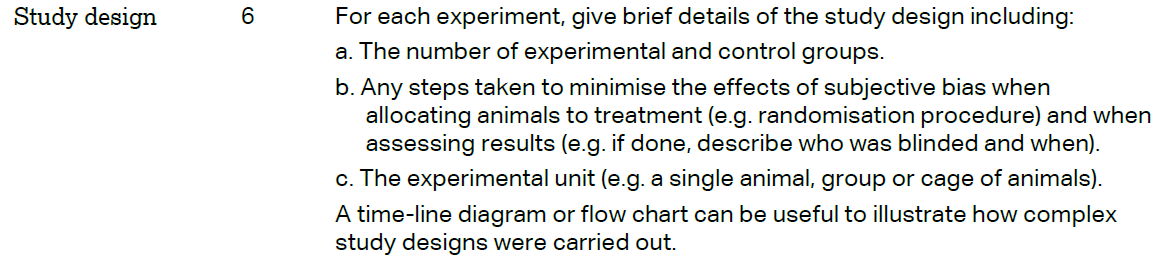 | | | Paragraph 15  Paragraph 15  Paragraph 15  Figure 3 |  |
| 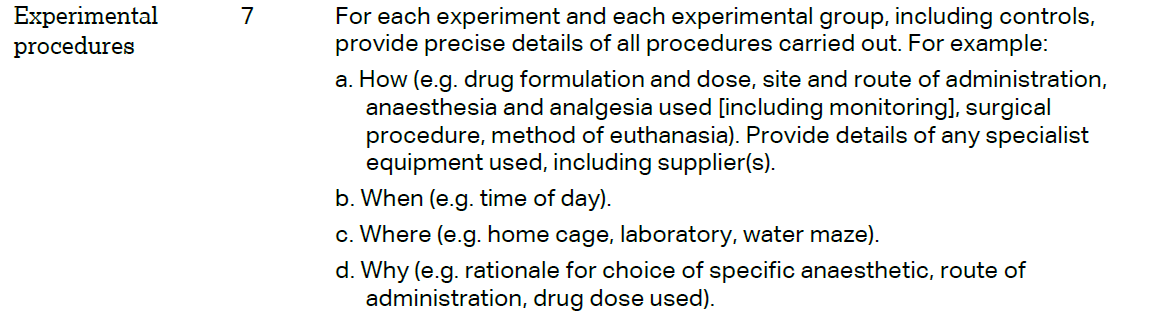 | | | Paragraphs 1, 15-19 |  |
| 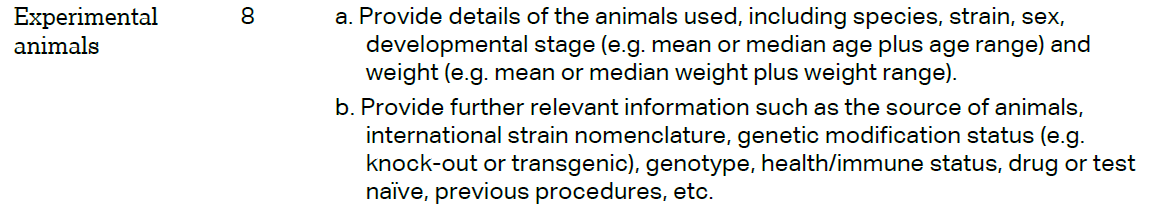 | | | Paragraph 15 |  |

The ARRIVE guidelines. Originally published in *PLoS Biology*, June 2010^1^

| 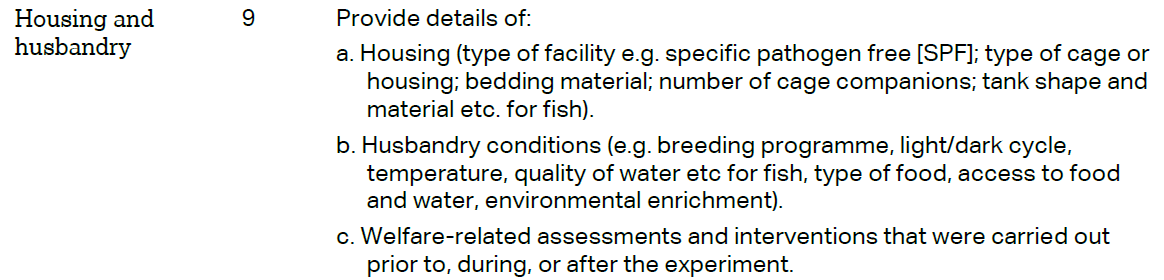 | Paragraph 15 | |
| --- | --- | --- |
| 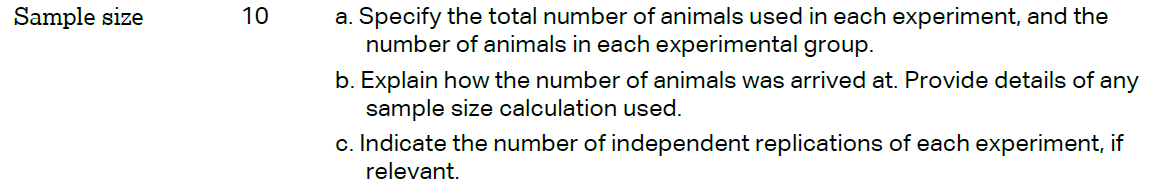 | Paragraph 15 | |
| 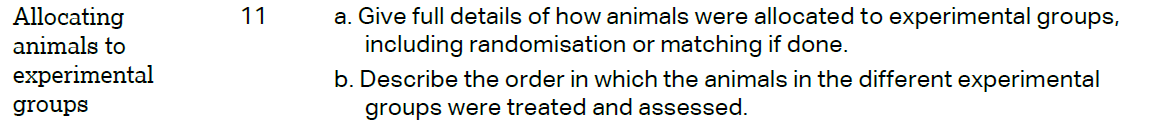 | Paragraph 15 | |
| 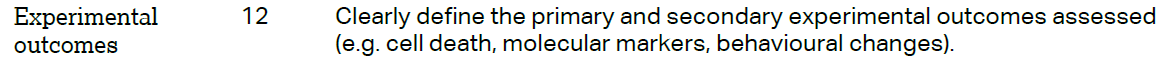 | Paragraph 1 | |
| 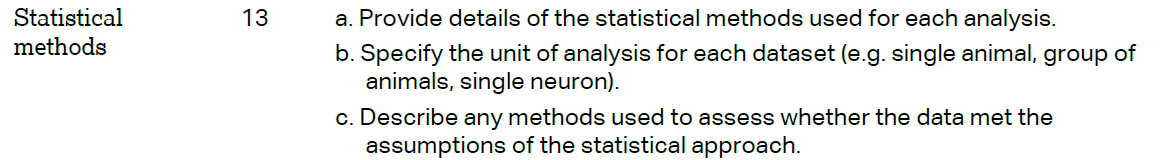 | Paragraph 20 | |
| RESULTS |  | |
| 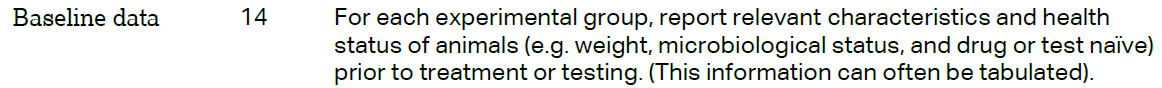 | Methods 1 | |
| 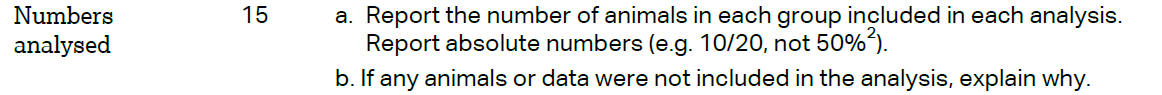 | Methods 15 | |
| 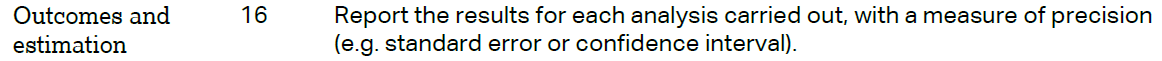 | Paragraphs 7-15  Figure4-6 | |
| 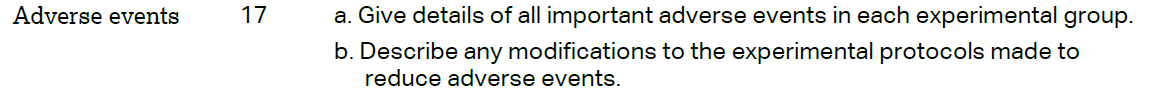 | Methods 1 | |
| DISCUSSION |  | |
| 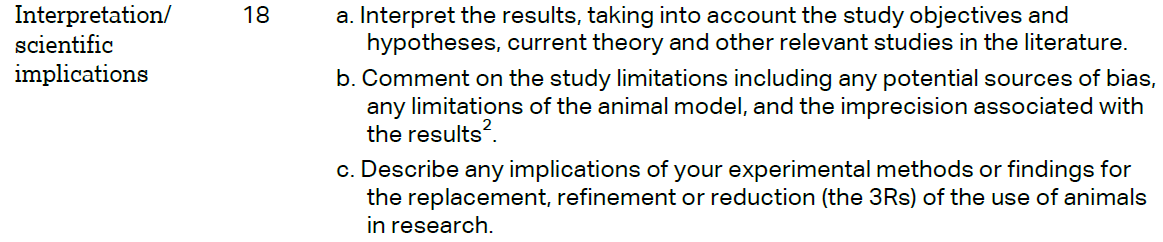 | Throughout | |
| 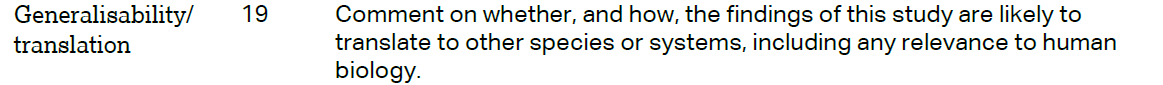 | Paragraphs 4-7 | |
| 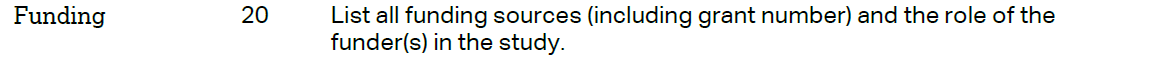 | | Acknoledgement |


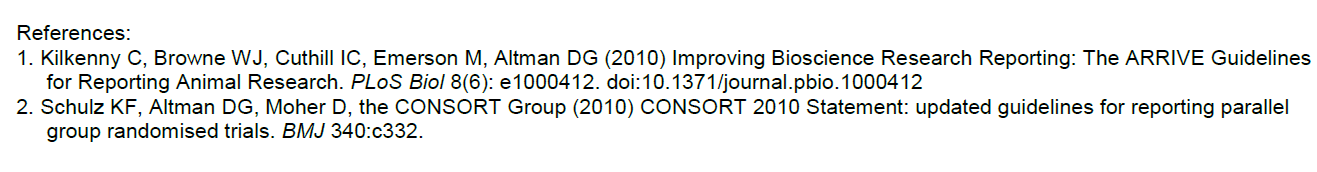

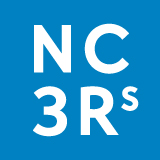

Supplement: S1 Checklist — (DOCX) [file pone.0196564.s001.docx]
