# Supplementary figures and images for: Development of experimental GBS vaccine for mucosal immunization
Source: PLoS One. 2018 May 4;13(5):e0196564. doi: 10.1371/journal.pone.0196564 (PMC5935385; doi:10.1371/journal.pone.0196564)

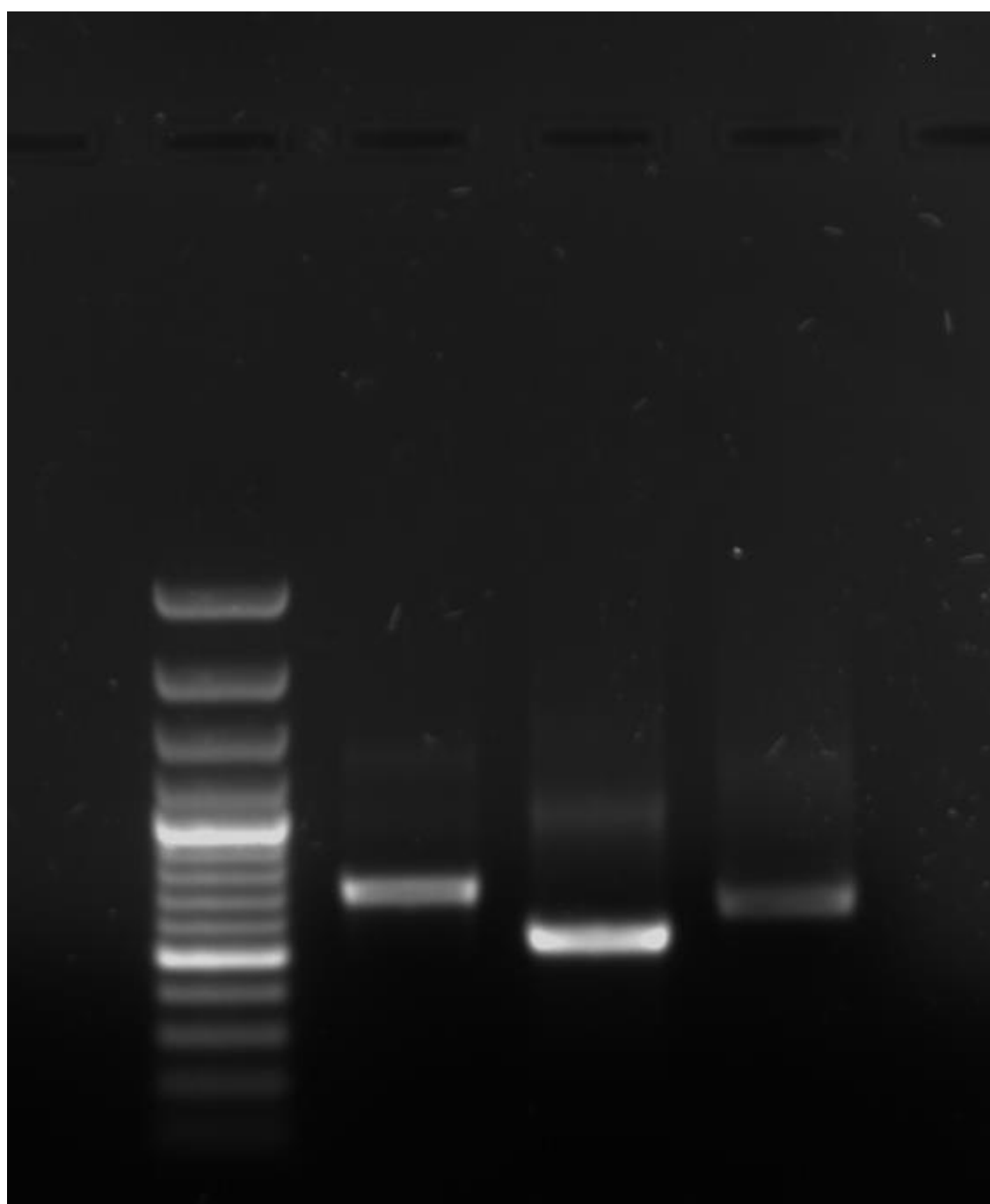

1

2

3

4

Supplement: S1 Fig — 1–100 bp Ladder DNA marker (100–3000 bp); 2 –the PCR product with the primers A1 and B1; 3 –the PCR product with the primers C1 and D1; 4 –the PCR product with the primers E1 and F1. (PDF) [file pone.0196564.s002.pdf]

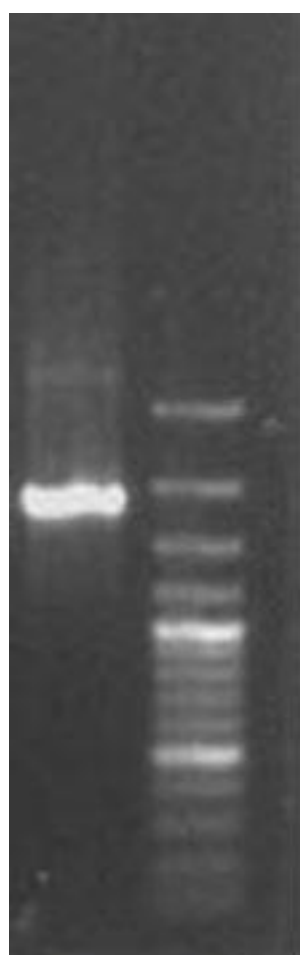

1

2

Supplement: S2 Fig — 1—PCR Product («fused» gene) with the primers A1 and D2; 2–100 bp Ladder DNA marker (100–3000 bp). (PDF) [file pone.0196564.s003.pdf]

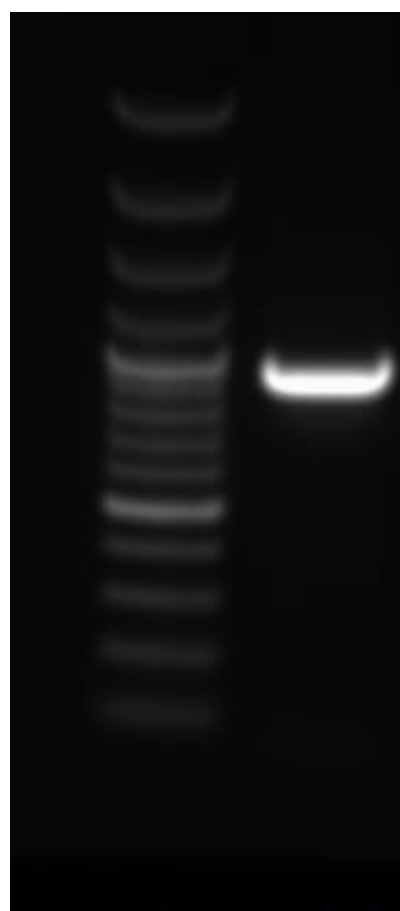

1

2

Supplement: S3 Fig — 1–100 bp Ladder DNA marker (100–3000 bp); 2—PCR product with primers B2 and B5 (PDF) [file pone.0196564.s004.pdf]
