# Supplementary material for: Development of experimental GBS vaccine for mucosal immunization
Source: PLoS One. 2018 May 4;13(5):e0196564. doi: 10.1371/journal.pone.0196564 (PMC5935385; doi:10.1371/journal.pone.0196564)
Supplement: S4 Fig — Primer B2 corresponds to the E.faecium chromosomal DNA outside the integrative plasmid. Primer B5 corresponds to the streptococcal Bac protein gene. (PDF) [file pone.0196564.s005.pdf]

D2-1

- **TGAGTGAACCACAGCCAGAA**ATTAATTCAAAAATGAGATCGATGAGAGCAGCTGG
- TATTGAGTTGAATGATACATTTCTATCTATTTACAGTTTAAATGGACAGTATCAG
- CAACGTGTGTCTTGGTATAATGACAATAATGAATCTGTCGGTGAACGTAATATTG
- ATATGAGAGAATTTGTTGGGTATGAAAAAATGGGTAGCTTACCTTATTTTGTCAC
- AACAGATACAGCATGTGCAGAATACAAAGCTCCTGCGTTATCAACAAACAATTTA
- ACTTCAAAAGTAGTGGGAGGACGTGCAGAAAAGGCTTATAGCTCGAATGATCATT
- TCACCGATGTTGTAGGAGCTGATACTTATCACAGAAGTGGTGTAACGTATACGCT
- TCAAGGCGCTTCCCCAACATTCATGATTGGCGCAAATACGAATAGTATGATGTTT
- AGCTTTGATACTGCATTGCTATGGACACCACAACCATCGAAGCCTACAAAAGAAG
- TGTTTAACAAAGCTAATACTGAAGAGGCAGCACACAATATTGACAAAAAAGTGAT
- TCCACAAGGATCAGATGTTTACTATCATATTCATCAAAAGTTTGATGCATTAACA
- GTCAACACAATGAACAAATACAAATCATTTAAAATCACTGATACCTTTGACAGCA
- **bac**
- AAAATTTTGATATGGTATCGGATGGGAAAAACTATGATGGCGCATTGTTTGATGA
- AACAAATGATTCTGATGCATTATTAGAATTAGAAAATCAATTTAACGAAACTAAT
- AGACTGTTACACATCAAACAACATGAAGAAGTTGAGAAAGATAAGAAAGCTAAGC
- AACAGAAAACTCTGAAACAGTCAGATACGAAAGTAGATCTAAGCAATATTGACAA
- AGAGCTTAATCATCAAAAAAGTCAAGTTGAAAAAATG**GCAGAGCAAAAGGGAATC**
- **AC**
