# Supplementary material for: Development of experimental GBS vaccine for mucosal immunization
Source: PLoS One. 2018 May 4;13(5):e0196564. doi: 10.1371/journal.pone.0196564 (PMC5935385; doi:10.1371/journal.pone.0196564)
Supplement: S1 Protocol — (PDF) [file pone.0196564.s006.pdf]

S1 Protocol. Conversion of antibody dilutions and OD values (based on ELISA readings) to antibody concentrations.

In order for the ELISA assay to be quantitative two special standard curves were made.

Serial two-fold dilutions of standard murine IgA or IgG were absorbed for night at 4°C on the plate bottom and then detected with HRP-linked appropriate secondary antibodies. OD values of the plate wells was measured and standard curves were generated by graphing the mean absorbance for each sample vs. the standard concentration. On each standard curve we choosed the portion that is linear and use that range of standard IgA and IgG concentrations to make standard curves for every experiment and each plate.

To quantify the concentration of antibodies in samples in indirect ELISA we absorbed Bac antigen on the bottom of plastic plates for night at 4°C. Simultaneously, we absorbed standard murine IgA or IgG in appropriate serial two-fold dilutions on each plate. Then we prepared serial two-fold dilutions of each sample, filling wells of standard curves with PBS. Then we made indirect ELISA according to the manufacturer's recommendations, using all wells. We choose the linear portion of the sample titration curve and calculated antibodies concentration comparing samples OD values with OD values of standard curve.

This approach was not appropriate for the detection of the absolute concentration of the immunoglobulins. However, it was useful for comparative evaluation of the dynamics of vaccination
